# Association of Surgical Timing with Outcomes in Early Stage Lung Cancer

**DOI:** 10.1007/s00268-023-06913-w

**Published:** 2023-01-25

**Authors:** Kian C. Banks, Jennifer R. Dusendang, Julie A. Schmittdiel, Diana S. Hsu, Simon K. Ashiku, Ashish R. Patel, Lori C. Sakoda, Jeffrey B. Velotta

**Affiliations:** 1 Department of Surgery, UCSF East Bay, 1411 E 31St St, Oakland, CA 94602 USA; 2 Department of Thoracic Surgery, Kaiser Permanente Northern California, 3600 Broadway, Oakland, CA 94611 USA; 3 Division of Research, Kaiser Permanente Northern California, 2000 Broadway, Oakland, CA 94612 USA; 4 Department of Health Systems Science, Kaiser Permanente Bernard J. Tyson School of Medicine, 98 S. Los Robles Avenue, Pasadena, CA 91101 USA

## Abstract

**Background:**

Optimal time to surgery for lung cancer is not well established. We aimed to assess whether time to surgery correlates with outcomes.

**Methods:**

We assessed patients 18–84 years old who were diagnosed with stage I/II lung cancer at our integrated healthcare system from 2009 to 2019. Time to surgery was defined to start with disease confirmation (imaging or biopsy) prior to the surgery scheduling date. Outcomes of unplanned return to care within 30 days of lung cancer surgery, all-cause mortality, and disease recurrence were compared based on time to surgery before and after 2, 4, and 12 weeks.

**Results:**

Of 2861 included patients, 70% were over 65 years old and 61% were female. Time to surgery occurred in 1–2 weeks for 6%, 3–4 weeks for 31%, 5–12 weeks for 58%, and 13–26 weeks for 5% of patients. Patients with time to surgery > 4 (vs. ≤ 4) weeks had greater risk of both death (hazard ratio (HR) 1.18, 95% confidence interval (CI) 1.00–1.39) and recurrence (HR 1.33, 95% CI 1.10–1.62). Associations were not statistically significant when dichotomizing time to surgery at 2 or 12 weeks for death (2 week HR 1.23, 95% CI 0.93–1.64; 12 week HR 1.35, 95% CI 0.97–1.88) and recurrence (2 week HR 1.54, 95% CI 0.85–2.80; 12 week HR 2.28, 95% CI 0.80–6.46).

**Conclusions:**

Early stage lung cancer patients with time to surgery within 4 weeks experienced lower rates of recurrence. Optimal time to surgical resection may be shorter than previously reported.

## Introduction

The time point at which delays to treatment lead to worse patient outcomes in lung cancer is not entirely clear [1–5]. Some have suggested worsened outcomes with various degrees of delays, though other evidence suggests that outcomes of patients with particularly short times to surgery may suffer due to rushed preoperative assessments [6–12]. While prior studies have identified time cutoffs to surgery for lung cancer beyond which patients experience adverse outcomes, these studies examined either small patient cohorts or data gathered from large databases in which time from diagnosis to treatment was not consistently defined [1–12]. For example, in many cases, date of diagnosis drawn from a national database is the same as the date of surgical resection (the time of tissue diagnosis). These studies also report only on overall survival and do not assess disease recurrence— a more specific finding related to lung cancer treatment. While the specific timing to definitive treatment may not be entirely clear, a systematic review suggests that expedient time to surgery does seem to convey favorable outcomes among patients with early stage lung cancer [1–13].

In 2021, Heiden et al. [14] published a Veterans Health Administration-based study in which they found worse survival and increased risk of recurrence among Veterans undergoing surgery more than 12 weeks after diagnosis of stage I non-small cell lung cancer (NSCLC). Notably, the authors attempted to standardize the measure of time to surgery (TTS) commencing from most recent suspicious abnormal computed tomography (CT) scan to date of surgery. Neither the Veterans Affairs hospital system nor its patients, however, are necessarily representative of those outside of that system. In this study, 96% of patients were male and average TTS was 10 weeks, which is likely longer than that at many centers [14]. The optimal interval from diagnosis of early stage lung cancer to surgery, therefore, remains a topic of interest.

In this study, we aimed to evaluate the association of TTS with outcomes among patients within a large integrated health system undergoing surgical resection for early stage lung cancer. We hypothesized that shorter TTS, measured as the interval from confirmatory imaging or biopsy prior to the operation scheduling date to surgical resection, would correlate with improved outcomes.

## Material and methods

### Setting

In 2015, Kaiser Permanente Northern California (KPNC) implemented centers of excellence (COE) for lung cancer care, reducing the number of facilities that performed lung cancer surgery from 16 to 5 in an effort to promote standardization and specialization of care [15, 16]. Lung cancer diagnosis and treatment consist of a multidisciplinary review board that meets weekly across all medical centers between radiology, pulmonology, oncology, and thoracic surgery. After the diagnosis of stage I or II lung cancer is suspected upon imaging with or without biopsy, the patient and surgeon discuss treatment options. More than 98% of patients undergo pulmonary function testing. Once the surgeon and patient decide to proceed with surgery, designated surgery schedulers determine the earliest available time. All procedures performed in studies involving human participants were in accordance with the ethical standards of the institutional research committee and with the 1964 Helsinki Declaration and its later amendments or comparable ethical standards. The study was approved by the institutional review board of KPNC (IRB: 1,610,759-1) and individual consent was waived because the risk to patients was deemed minimal.

### Study design and population

This retrospective cohort study consists of patients aged 18–84 years who underwent elective surgery for stage I or II lung cancer between 1/1/2009 and 12/31/2019 who also had a CT or positron emission tomography (PET) scan of the lungs in the 6 months prior to surgery. Patients were identified using our regional cancer registry, which adheres to data standards of the Surveillance, Epidemiology, and End Results (SEER) program. We defined TTS as the time period from the confirmatory testing date (imaging or biopsy) prior to scheduling of surgery to the surgery date. We believe this is a more standardized definition given that some, but not all, patients with suspicious lesions undergo preoperative biopsy in our system. The last biopsy or scan prior to the surgery scheduling date was chosen as the start point for the TTS interval as this was deemed the point at which surgeons had the necessary staging and diagnostic information to confirm high likelihood of stage I or II lung cancer and therefore recommended surgery. Eligible scans of the lungs included CT with or without intravenous contrast, CT angiography, CT guidance for biopsies, or PET scans. Fleischner Society criteria for pulmonary nodules are used with weekly evaluation by a multidisciplinary panel of radiologists, pulmonologists, thoracic surgeons, and oncologists to define suspicious pulmonary nodules [17].

Patients with less than one year of prior health plan membership were excluded to ensure capture of comorbidities, and patients with prior documented lung cancer were excluded. In accordance with National Comprehensive Cancer Network recommendations, our early stage lung cancer patients proceed directly to surgery [18]. Hence, both stage I and II disease were included to be inclusive of all disease in which upfront surgery is performed. Patients downstaged to stage I or II disease with neoadjuvant chemotherapy in the year prior to surgery were excluded.

We assessed three outcomes: unplanned return to care, all-cause mortality, and recurrence. Unplanned return to care included any emergency room visits, readmissions, or reoperations in the 30 days following lung cancer surgery. Death information was available using an internal database which aggregates data on vital status from KPNC hospitals, social security files, California state death registry, and the National Death Index. We followed patients from the date of surgery until the first of death (outcome), disenrollment from the health plan, the end of data availability on 12/31/2019, or the end of 5 years of follow-up. Recurrence information was collected from the KPNC cancer registry which began systematically collecting cancer recurrence data in 2016. Therefore, we only assessed recurrence as an outcome for our lung cancer cases that were diagnosed 2016–2019.

The main exposure of interest was TTS, as defined above. Based on prior research and our own internal data, we assessed three separate cut-points for TTS, 2 weeks (≤ 4 days), 4 weeks (≤28 days), and 12 weeks (≤ 4 days) [8, 14, 19]. The 2-week cut-point was chosen based off our institution’s internal quality benchmarks set prior to the study start date which target a time from surgical consultation to surgery of less than 2 weeks. This is also in line with the Cancer Care Ontario guidelines for cancer surgery [19]. The 4-week cut-point was chosen based on our own institution’s initial feasibility data which is similar to but shorter than the 38 days suggested by Yang et al.[8]. The 12-week cut-point was chosen based on the previously mentioned study by Heiden et al. [14].

Other variables included in our models were patient age at surgery, sex, race/ethnicity, Charlson comorbidity score, diagnosis of non-lung cancer in the year prior to surgery, smoking history (ever vs never), cancer stage, histology, performance of mediastinoscopy, location, year, and hospital of surgery. Histology was grouped into adenocarcinoma, squamous cell, and other histologies. Year of surgery was separated into 2009–2014 and 2015–2019 based on the regionalization of COE for lung surgery occurring in 2015.

### Statistical analysis

Logistic regression was used to model the association of TTS with any unplanned return to care within 30 days of surgery. Cox proportional hazards modeling was used to model associations with death and recurrence. The models for recurrence were restricted to only surgeries performed from 2016 to 2019. All models adjusted for all of the covariables defined above. Hospital of surgery was included in all models as a random intercept (cluster variable) to account for variations in practice by hospital. The proportional hazards assumption was assessed for all cut-point values (2, 4, 12 weeks) in models for death and recurrence.

## Results

A total of 2861 patients met inclusion criteria. Baseline characteristics can be found in Tables 1,2. Of all patients, 52% were treated in the later time period, 61% were female, and 78% had stage 1 disease. Notably, while mediastinoscopy staging was performed in 10.9% of all patients, it was not associated with TTS when compared to patients without mediastinoscopy (*p* = 0.48). Also, while minimally invasive adenocarcinoma and bronchioalveolar carcinoma made up 5.70% of cases, there was no association with these subtypes and time to surgery compared to cases with other histologic subtypes (*p* = 0.17).

**Table 1:** Characteristics of patients undergoing surgery for stage 1–2 lung cancer, by time period

Characteristic	Overall *N *= 2861 N (%)	2009–2014 *N* = 1378 N (%)	2015–2019 *N* = 1483 N (%)
*Weeks from CT to surgery*			
1–2	174 (6)	91 (7)	83 (6)
3–4	879 (31)	464 (34)	415 (28)
5–12	1655 (58)	768 (56)	887 (60)
13–26	153 (5)	55 (4)	98 (7)
*Age, years*			
18–54	229 (8)	117 (8)	112 (8)
55–64	630 (22)	314 (23)	316 (21)
65–74	1214 (42)	560 (41)	654 (44)
75–84	788 (28)	387 (28)	401 (27)
*Sex*			
Female	1742 (61)	805 (58)	937 (63)
Male	1119 (39)	573 (42)	546 (37)
*Race/ethnicity*			
Asian	409 (14)	197 (14)	212 (14)
Black	201 (7)	81 (6)	120 (8)
Latinx	181 (6)	75 (5)	106 (7)
White	1877 (66)	924 (67)	953 (64)
Other	193 (7)	101 (7)	92 (6)
*Charlson comorbidity score*			
0–2	871 (30)	515 (37)	356 (24)
3–4	1011 (35)	466 (34)	545 (37)
5 +	979 (34)	397 (29)	582 (39)
*Other cancer in year prior*			
Yes	163 (6)	71 (5)	92 (6)
No	2698 (94)	1307 (95)	1391 (94)
*Smoking history*^*a*^			
Yes	2152 (75)	1068 (78)	1084 (73)
No	705 (25)	309 (22)	396 (27)
*Cancer stage*			
1	2241 (78)	1069 (78)	1172 (79)
2	620 (22)	309 (22)	311 (21)
*Cancer histology*			
Adenocarcinomas	2128 (74)	983 (71)	1145 (77)
Squamous cell	454 (16)	232 (17)	222 (15)
Other	279 (10)	163 (12)	116 (8)
*Cancer site*			
Upper lobe	1630 (57)	806 (58)	824 (56)
Middle lobe	189 (7)	88 (6)	101 (7)
Lower lobe	989 (35)	457 (33)	532 (36)
Other	53 (2)	27 (2)	26 (2)

CT, computed tomography. ^a^4 patients had missing smoking history information

**Table 2:** Characteristics of patients undergoing surgery for stage 1–2 lung cancer, by 4-week time cut-point

Characteristic	Overall, *N* = 2861 N (%)	Weeks from CT to surgery	
		≤ 4 N = 1053	> 4 N = 1808 N (%)
*Year of surgery*			
2009–2014	1378 (48)	555 (53)	823 (46)
20,152,019	1483 (52)	498 (47)	985 (54)
*Age*,* years*			
18–54	229 (8)	99 (9)	130 (7)
55–64	630 (22)	243 (23)	387 (21)
65–74	1214 (42)	438 (42)	776 (43)
75–84	788 (28)	273 (26)	515 (28)
*Sex*			
Female	1742 (61)	633 (60)	1109 (61)
Male	1119 (39)	420 (40)	699 (39)
*Race*/*ethnicity*			
White	1877 (66)	684 (65)	1193 (66)
Asian	409 (14)	157 (15)	252 (14)
Black	201 (7)	74 (7)	127 (7)
Latinx	181 (6)	60 (6)	121 (7)
Other	193 (7)	78 (7)	115 (6)
*Charlson comorbidity score*			
0–2	871 (30)	317 (30)	554 (31)
3–4	1011 (35)	381 (36)	630 (35)
5 +	979 (34)	355 (34)	624 (35)
*Other cancer in year prior*			
No	2698 (94)	988 (94)	1710 (95)
Yes	163 (6)	65 (6)	98 (5)
*Smoking history*^*a*^			
No	705 (25)	271 (26)	434 (24)
Yes	2152 (75)	781 (74)	1371 (76)
*Mediastinoscopy*			
No	2548 (89)	936 (89)	1612 (89)
Yes	313 (11)	117 (11)	196 (11)
*Cancer stage*			
1	2241 (78)	832 (79)	1409 (78)
2	620 (22)	221 (21)	399 (22)
*Cancer histology*			
Adenomas/adenocarcinomas	2128 (74)	784 (74)	1344 (74)
Other	279 (10)	101 (10)	178 (10)
Squamous cell	454 (16)	168 (16)	286 (16)
*Minimally invasive adenocarcinoma*			
No	2698 (94)	1001 (95)	1697 (94)
Yes	163 (6)	52 (5)	111 (6)
*Cancer site*			
Upper lobe	1630 (57)	606 (58)	1024 (57)
Lower lobe	989 (35)	366 (35)	623 (34)
Middle lobe	189 (7)	66 (6)	123 (7)
Other	53 (2)	15 (1)	38 (2)

CT, computed tomography.^a^ 4 patients had missing smoking history information

**Fig. 1:**
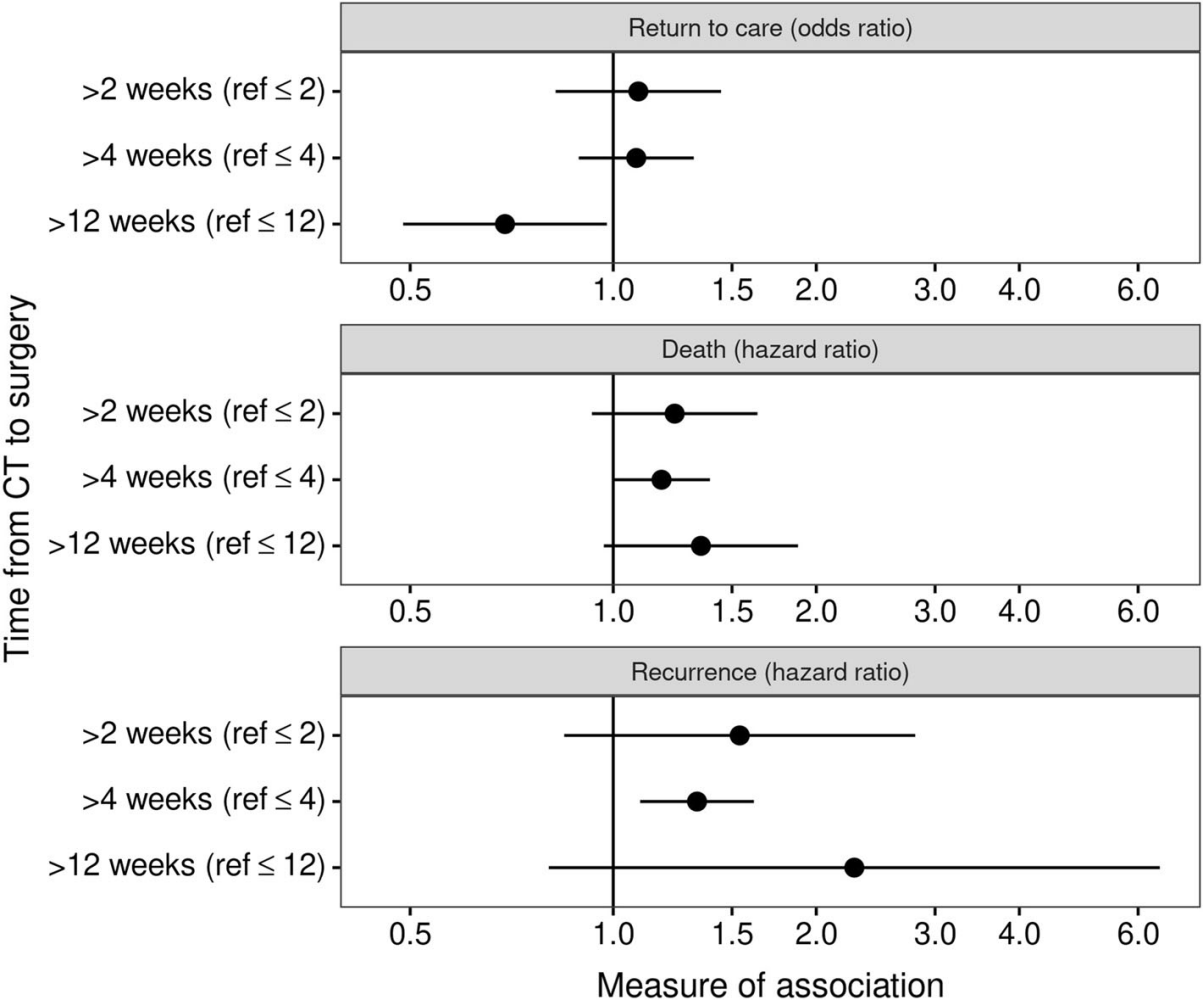
Adjusted odds ratios for unplanned return to care and hazard ratios for death and recurrence associated with time to surgery dichotomized using different cut-points. Ref, reference; CT, computed tomography. Each line in the figure represents the 95% confidence interval for each model. All models are adjusted for patient age, sex, race/ethnicity, Charlson comorbidity score, diagnosis of non-lung cancer in the prior year, stage, histology, and site. Models for unplanned return to care within 30 days and death during follow-up also adjust for year of surgery. Year of surgery was not included in the models for recurrence because recurrence data only started being collected systematically in 2016

In adjusted logistic regression models, patients with TTS greater than 12 weeks had decreased odds of unplanned return to care within 30 days compared to patients with TTS 12 weeks or shorter (odds ratio (OR) 0.69, 95% confidence interval (CI) 0.49–0.98) (Fig. 1, Table 3). Models with cut-points at 2 (OR 1.09, 95% CI 0.82–1.44) or 4 weeks (OR 1.08, 95% CI 0.89–1.32) did not have statistically significant values for an association between TTS and unplanned return to care. Patients with TTS greater than 4 weeks had an increased rate of both death (HR 1.18, CI 1.00–1.39) and recurrence (HR 1.33, 1.10–1.62). Though not statistically significant, the results for 2- and 12-week cut-points did follow the same pattern with patients with longer TTS trending toward higher rates of death and recurrence (2-week cut-point for death model HR 1.23, CI 0.93–1.64; 12-week cut-point for death model HR 1.35, CI 0.97–1.88; 2-week cut-point for recurrence model HR 1.54, 0.85–2.80; 12-week cut-point for recurrence model HR 2.28, CI 0.80–6.46). Of all deaths, 73.7% were due to lung cancer.

**Table 3:** Adjusted odds ratios and hazard ratios from 9 adjusted models

Time from CT to surgery	Unplanned return to care within 30 days		Death during follow-up		Recurrence during follow-up(2016–2019 cases only)	
	# events/N	OR (95% CI)	# events/N	HR (95% CI)	# events/N	HR (95% CI)
*Model 1*						
> 2 weeks	624/2687	1.09 (0.82–1.44)	503/2687	1.23 (0.93–1.64)	110/1146	1.54 (0.85–2.80)
≤ 2 weeks	37/174	Ref	28/174	Ref	4/55	Ref
*Model 2*						
> 4 weeks	428/1808	1.08 (0.89–1.32)	338/1808	1.18 (1.00–1.39)	80/836	1.33 (1.10–1.62)
≤ 4 weeks	233/1053	Ref	193/1053	Ref	34/365	Ref
*Model 3*						
> 12 weeks	26/153	0.69 (0.49–0.98)	28/153	1.35 (0.97–1.88)	11/85	2.28 (0.80–6.46)
≤ 12 weeks	635/2708	Ref	503/2708	Ref	103/1116	Ref

CT, computed tomography; CI, confidence interval; Ref, reference

All models are adjusted for patient age, sex, race/ethnicity, Charlson comorbidity score, smoking history, having another non-lung cancer diagnosed in the year prior, cancer stage, cancer histology, and cancer site. Models predicting unplanned return to care within 30 days and death during follow-up also adjust for year of surgery. Year of surgery was not included in the model predicting recurrence because recurrence data only started being collected systematically in 2016

Average lengths of follow-up among patients in the 2009–2014 and 2015–2019 cohorts who died were 4.0 and 2.2 years, respectively. Patients had average follow-up for recurrence of 1.7 years with a maximum follow-up of 4 years and an interquartile range of 0.74–2.6 years.

In addition to TTS, other variables were associated with our outcomes of interest (Table 4). Black patients and patients with middle or lower-lobe cancer had reduced odds of having an unplanned return to care within 30 days, while patients with smoking history or squamous cell histology were more likely to have an unplanned return to care. Black patients were also less likely than White patients to die during follow-up, although Black patients had a slightly shorter average length of follow-up (2.8 years vs 3.1 years). Higher rates of recurrence were found in Asian (HR 2.13, CI 1.46–3.11) and Latinx (HR 1.26, CI 1.01–1.57) patients.

**Table 4:** Results from fully adjusted models for outcomes associated with time from CT to surgery using a 4-week cut-point

Characteristic	Unplanned return to care within 30 days		Death during follow-up		Recurrence during follow-up (2016–2019 cases only)	
	% with outcome	OR (95% CI)	Incidence per 100 person-years	HR (95% CI)	Incidence per 100 person-years	HR (95% CI)
*Time from CT to surgery*						
> 4 weeks	24	1.08 (0.89–1.32)	6.5	1.18 (1.00–1.39)	6.1	1.33 (1.10–1.62)
≤ 4 weeks	22	1.00 (Ref.)	5.4	1.00 (Ref.)	4.5	1.00 (Ref.)
*Surgery year*						
2009-2014^a^	23	0.93 (0.78–1.10)	6.9	1.50 (1.20–1.88)	-	-
2015–2019	24	1.00 (Ref.)	4.6	1.00 (Ref.)	5.5	-
*Age*						
18–54	23	1.00 (Ref.)	1.6	1.00 (Ref.)	3.7	1.00 (Ref.)
55–64	20	0.71 (0.49–1.03)	4.2	2.51 (1.13–5.58)	5.4	1.31 (0.78–2.21)
65–74	23	0.79 (0.51–1.22)	5.6	3.04 (1.44–6.42)	6.1	1.60 (0.98–2.60)
75–84	26	0.90 (0.51–1.41)	9.8	4.99 (2.45–10.1)	5.2	1.26 (0.77–2.06)
*Sex*						
Female	23	1.00 (Ref.)	5.4	1.00 (Ref.)	5.0	1.00 (Ref.)
Male	24	0.97 (0.77–1.22)	7.1	1.12 (0.98–1.29)	6.5	1.13 (0.63–2.01)
*Race*/*ethnicity*						
Asian	19	0.86 (0.70–1.07)	5.2	1.21 (0.99–1.47)	8.1	2.13 (1.46–3.11)
Black	20	0.76 (0.60–0.96)	4.4	0.76 (0.60–0.95)	9.3	1.91 (0.79–4.65)
Latinx	22	0.96 (0.64–1.43)	6.4	1.20 (0.88–1.63)	5.7	1.26 (1.01–1.57)
White	24	1.00 (Ref.)	6.3	1.00 (Ref.)	4.6	1.00 (Ref.)
Other	28	1.35 (1.13–1.61)	6.2	1.05 (0.83–1.34)	4.4	1.00 (0.39–2.59)
*Charlson comorbidity score*						
0–2	21	1.00 (Ref.)	4.0	1.00 (Ref.)	4.3	1.00 (Ref.)
3–4	24	1.14 (0.95–1.37)	6.1	1.30 (1.02–1.66)	4.6	0.97 (0.57–1.43)
5 +	25	1.13 (0.88–1.44)	8.4	1.58 (1.30–1.91)	7.3	1.40 (0.83–2.37)
*Other cancer in year prior*						
Yes	21	0.89 (0.61–1.30)	7.3	1.18 (0.82–1.69)	5.4	0.97 (0.56–1.69)
No	23	1.00 (Ref.)	6.0	1.00 (Ref.)	5.5	1.00 (Ref.)
*Smoking history*						
Yes	25	1.43 (1.18–1.73)	7.0	1.76 (1.34–2.33)	6.0	1.45 (0.97–2.16)
No	18	1.00 (Ref.)	3.1	1.00 (Ref.)	4.3	1.00 (Ref.)
*Cancer stage*						
1	22	1.00 (Ref.)	5.2	1.00 (Ref.)	4.3	1.00 (Ref.)
2	26	1.16 (0.89–1.52)	9.6	1.69 (1.46–1.96)	11.2	2.56 (1.89–3.47)
*Cancer histology*						
Adenocarcinoma	22	1.00 (Ref.)	4.9	1.00 (Ref.)	5.2	1.00 (Ref.)
Squamous cell	30	1.32 (1.11–1.58)	8.5	1.25 (0.88–1.77)	5.4	0.82 (0.61–1.09)
Other	23	0.98 (0.74–1.31)	11.0	1.97 (1.53–2.53)	10.7	2.00 (0.96–4.17)
*Cancer site*						
Upper lobe	25	1.00 (Ref.)	6.1	1.00 (Ref.)	6.0	1.00 (Ref.)
Middle lobe	17	0.65 (0.45–0.92)	4.4	0.85 (0.52–1.41)	7.2	1.35 (0.84–2.17)
Lower lobe	21	0.81 (0.70–0.94)	6.2	1.05 (0.79–1.40)	4.6	0.73 (0.43–1.23)
Other	28	1.21 (0.49–2.97)	5.5	0.85 (0.58–1.24)	2.8	0.43 (0.14–1.32)

CI, confidence interval; CT, computed tomography; Ref, reference

^a^year was not included in the model predicting recurrence because the cancer registry data are only complete for 2016–2019 cases

Of the 2861 patients, 76.8% underwent lobectomy. Given this large majority, we performed a sub-group analysis of these patients at the 4-week time cut-point. We found similar results to our overall results with significantly increased hazard ratios for death and recurrence (1.17, CI 1.00–1.38 and 1.34, CI 1.09–1.65, respectively).

## Discussion

Our study suggests that TTS longer than 4 weeks is associated with increased rate of recurrence and death.

The use of confirmatory testing prior to surgery scheduling as a starting point allows for a standardized definition for TTS. In 2008, Gould et al*.* [1] used the concept of interval between imaging and treatment initiation to define time to treatment; however, they used the initial chest radiograph or CT scan in which a suspicious finding was later confirmed to be lung cancer. We believe this designated interval does not allow for the same degree of standardization for comparison. Suspicious findings may undergo various time intervals of lung nodule surveillance or workup prior to the decision to biopsy or treat, and evidence suggests that this time period of evaluation for unclear imaging findings may not have a bearing on patient outcome as long as the patient maintains continuity of care [20, 21].

Our study also avoids the potential systematic bias in which tissue diagnosis alone defines the beginning of the TTS interval. In these cases, if tissue diagnosis is performed at the time of surgery, TTS is zero. This aberrantly affects any comparison to TTS for patients with tissue diagnosis prior to surgery. Additionally, Tang et al. [22] found that patients with tissue diagnosis at the time of surgery generally had smaller tumors and were better surgical candidates than those with prior tissue diagnoses emphasizing that these are not comparable groups.

Our study shares similarities to that by Heiden et al*.* [14] in that both examine a large patient population, define a standardized interval prior to surgery to define TTS, and find that a delay in TTS negatively impacts risk of recurrence and mortality. Our study, however, includes a population more generalizable to the population at large, includes more patients treated within 4 weeks, and, critically, finds that the TTS at which patients experience worse outcomes begins at 4 weeks rather than 12. Additionally, Heiden et al. [14] do not account for patients who undergo preoperative biopsy, so despite their attempts at standardization, some patients may have undergone preoperative biopsy while others went directly to surgery. By using either biopsy or last imaging, our definition of TTS further standardizes this time interval and provides a more comparable starting point.

The TTS within our system is affected by our internal standards process, prompt access to virtual preoperative anesthesia evaluation appointments, and ability to quickly book operative cases with a multitude of surgery schedulers in each surgical department. Also, our system allows for quick transfer of information via a standardized staff messaging system integrated for all of KPNC, thus minimizing delays for physicians and staff when accessing imaging, discussing patient information, and communicating with patients. Our internal standards emphasizing expedient surgical scheduling likely play a role in why mediastinal staging via mediastinoscopy is not associated with delays to surgery.

The finding that TTS shorter than 2 weeks does not improve outcomes may occur for several reasons. It is possible that the evaluation and medical optimization process which may consist of further cardiopulmonary testing prior to surgery may have been rushed in a subset of these patients or omitted completely due to time constraints. It could also be that features on the imaging for some patients were more aggressive in appearance prompting the surgeon to opt for earlier surgical intervention for more aggressive disease. Also, given the range of the confidence intervals of this group’s outcomes, some patients may have benefited from the shorter TTS while others may not have. If this is the case, identifying these patients would provide an area for further improvement. Additionally, there was a much smaller number of patients with surgery within 2 weeks, so our findings may also be due to reduced statistical power for this group.

The finding that a TTS shorter or longer than 12 weeks was not associated with survival or recurrence is interesting given the prior literature. Only 5% of our patients had TTS longer than 12 weeks and the variability in the confidence intervals of all three outcomes in this group was also relatively large. It is possible that some of these patients would have benefitted from earlier surgery while others would not have. Specific features on imaging were not collected, but it is possible that some of these patients had features that, while concerning enough to prompt surgery, were less aggressive in appearance. Interestingly, patients with TTS longer than 12 weeks were the only ones that were less likely to have an unplanned return to care within 30 days of surgery, perhaps due to increased time for medical optimization leading to less complicated post-operative recoveries. Additionally, characteristics that may appear less aggressive on imaging likely include smaller size, more peripheral location, and more ground glass component rather than solid. Such tumors may require less technically complicated dissections and, in some cases, less lung being resected with subsequent lower likelihood for post-operative issues that would lead to an unplanned return to care within 30 days.

While preoperative workup and comorbidity status may influence TTS and death, the distribution of Charlson comorbidity scores was similar between patients with TTS ≤  and  > 4 weeks. Also, the vast majority of deaths were related directly to lung cancer.

Notably, our study included 61% females which contrasts with the 3.7% included in the study by Heiden et al*.* [14] and broadens the generalizability of our results. Also, Black patients experienced reduced odds of unplanned return to care within 30 days and had reduced risk of death during follow-up compared to White patients. Asian and Latinx patients had increased risk of recurrence compared to White patients. These findings warrant further investigation.

Of note, the group prior to regionalization experienced higher mortality, but, due to the study end-point at the end of 2019, the average follow-up duration was nearly twice as long in this group.

Given that lobectomy is commonly used for definitive resection of early stage lung cancer, the sub-group analysis of only these patients further validates our results at the 4-week cut-point and allows for a more granular interpretation of our data. We believe this allows our results to be more easily translatable to clinical practice.

There are a few additional limitations to our study. Given the retrospective design, we cannot definitively rule out the possibility of unmeasured confounders leading to the associations observed. Also, while this TTS definition allows for improved standardization and comparison among patient groups, it is not perfect. Comparison with this metric alone does not take into account more or less worrisome features on imaging that may affect how quickly a patient is scheduled for surgery such as nodule type, ground glass appearance, the presence of spiculations, location, and SUV. Such imaging characteristics along with histological characteristics such as tumor grade that may suggest aggressive lesions are likely treated with more urgency than those that appear more indolent. Additionally, our recurrence data were only available from 2016 to 2019, with a maximum follow-up for recurrence of four years. However, recurrence data are not available prior to 2016. Lastly, we do not have information on the exact reason initial imaging was performed for each case. We plan to further investigate this to distinguish whether imaging was incidental, conducted for screening, or prompted by symptoms.

Stage I and II lung cancer patients with TTS of less than or equal to 4 weeks experienced lower rates of recurrence and a trend toward decreased mortality during follow-up than those with TTS greater than 4 weeks. Expeditious workup without compromising optimization of comorbidities may play a role in improving outcomes among patients with early stage lung cancer.

## References

[1] Gould MK, Ghaus SJ, Olsson JK (2008). Timeliness of care in veterans with non-small cell lung cancer. Chest.

[2] Olsson JK, Schultz EM, Gould MK (2009). Timeliness of care in patients with lung cancer: a systematic review. Thorax.

[3] Kanarek NF, Hooker CM, Mathieu L (2014). Survival after community diagnosis of early-stage non-small cell lung cancer. Am J Med.

[4] Vinod SK, Chandra A, Berthelsen A (2017). Does timeliness of care in non-small cell lung cancer impact on survival?. Lung Cancer.

[5] Ha D, Ries AL, Montgrain P (2018). Time to treatment and survival in veterans with lung cancer eligible for curative intent therapy. Respir Med.

[6] Samson P, Patel A, Garrett T (2015). Effects of delayed surgical resection on short-term and long-term outcomes in clinical stage i non-small cell lung cancer. Ann Thorac Surg.

[7] Gomez DR, Liao K-P, Swisher SG (2015). Time to treatment as a quality metric in lung cancer: staging studies, time to treatment, and patient survival. Radiother Oncol.

[8] Yang C-FJ, Wang H, Kumar A (2017). Impact of timing of lobectomy on survival for clinical stage IA lung squamous cell carcinoma. Chest.

[9] Anggondowati T, Ganti AK, Islam KMM (2020). Impact of time-to-treatment on overall survival of non-small cell lung cancer patients—an analysis of the national cancer database. Transl Lung Cancer Res.

[10] Zhang L, Hsieh M-C, Rennert L (2021). Diagnosis-to-surgery interval and survival for different histologies of stage I-IIA lung cancer. Transl Lung Cancer Res.

[11] Mayne NR, Elser HC, Darling AJ (2021). Estimating the impact of extended delay to surgery for stage I non-small-cell lung cancer on survival. Ann Surg.

[12] Nadpara P, Madhavan SS, Tworek C (2015). Guideline-concordant timely lung cancer care and prognosis among elderly patients in the United States: a population-based study. Cancer Epidemiol.

[13] Hall H, Tocock A, Burdett S (2022). Association between time-to-treatment and outcomes in non-small cell lung cancer: a systematic review. Thorax.

[14] Heiden BT, Eaton DB, Engelhardt KE (2021). Analysis of delayed surgical treatment and oncologic outcomes in clinical stage I non-small cell lung cancer. JAMA Netw Open.

[15] Ely S, Jiang S-F, Patel AR (2020). Regionalization of lung cancer surgery improves outcomes in an integrated health care system. Ann Thorac Surg.

[16] Ely S, Jiang S-F, Dominguez DA (2021). Effect of thoracic surgery regionalization on long-term survival after lung cancer resection. J Thorac Cardiovasc Surg.

[17] MacMahon H, Naidich DP, Goo JM (2017). Guidelines for management of incidental pulmonary nodules detected on ct images: from the fleischner society 2017. Radiology.

[18] National Comprehensive Cancer Network. Non-Small Cell Lung Cancer (Version 3.2022). In: NCCN. https://www.nccn.org/professionals/physician_gls/pdf/nscl.pdf. Accessed 5 Apr 2022 10.6004/jnccn.2022.0025 35545176

[19] https://www.cancercareontario.ca/en/guidelines-advice/types-of-cancer/3211. In: Cancer Care Ont. https://www.cancercareontario.ca/en/guidelines-advice/types-of-cancer/3211. Accessed 7 Dec 2022

[20] Kashiwabara K, Koshi S, Ota K (2002). Outcome in patients with lung cancer found retrospectively to have had evidence of disease on past lung cancer mass screening roentgenograms. Lung Cancer.

[21] Kashiwabara K, Koshi S, Itonaga K (2003). Outcome in patients with lung cancer found on lung cancer mass screening roentgenograms, but who did not subsequently consult a doctor. Lung Cancer.

[22] Tang A, Ahmad U, Raja S (2021). How much delay matters? How time to treatment impacts overall survival in early stage lung cancer. Ann Surg.

